# Author Correction: Solution-sheared supramolecular oligomers with enhanced thermal resistance in interfacial adhesion and bulk cohesion

**DOI:** 10.1038/s41467-026-69146-0

**Published:** 2026-02-11

**Authors:** Gang Lu, Rui Ma, Yuanyuan Zhao, Dianyu Wang, Wentao Shang, Huaguo Chen, Shahid Ali Khan, Ming Li, Eduardo Saiz

**Affiliations:** 1 Department of Chemical and Biomolecular Engineering, University of Pennsylvania, Philadelphia, PA USA; 2 School of Energy and Environment, City University of Hong Kong, Hong Kong, China; 3 NTNU Nanomechanical Lab, Department of Structural Engineering, Norwegian University of Science and Technology, Trondheim, Norway; 4 School of Fashion and Textile, The Hong Kong Polytechnic University, Hong Kong, China; 5 School of Chemical Engineering, Zhengzhou University, Zhengzhou, China; 6 Energy and Electricity Research Center, International Energy College, Jinan University, Guangdong, China; 7 Department of Architecture and Civil Engineering, City University of Hong Kong, Hong Kong, China; 8 Centre of Advanced Structural Ceramics, Department of Materials, Imperial College London, London, UK

**Keywords:** Polymer synthesis, Self-assembly, Polymers

Correction to: *Nature Communications* 10.1038/s41467-025-63123-9, published online 20 August 2025

In the original version of this Article, details regarding the fitting procedure for the adhesion force distribution depicted in Fig. 4h and i were omitted. The Gaussian fitting was applied to illustrate the general trend in adhesion behavior rather than individual measurement fluctuations; the raw datasets contain experimental noise, but this does not affect the validity of the reported conclusions. In the corrected version, the sentence ‘Adhesion force distribution depicted in Fig. 4h and i was fitted using a Gaussian function’. Was added to the description of the structural characterization in the methods section of the publication. Additionally, the authors provide the source data for the adhesion force distribution. The sentence ‘Source data are provided with this paper’ has been added to the Data availability statement. This has been corrected in both the PDF and HTML versions of the Article.

