# Supplementary material for: Solution-sheared supramolecular oligomers with enhanced thermal resistance in interfacial adhesion and bulk cohesion
Source: Nat Commun. 2025 Aug 20;16:7754. doi: 10.1038/s41467-025-63123-9 (PMC12368072; doi:10.1038/s41467-025-63123-9)
Supplement: Supplementary file 1 — Supplementary Information [file 41467_2025_63123_MOESM1_ESM.pdf]

Supplementary Information

Solution-Sheared Supramolecular Oligomers with Enhanced Thermal Resistance in Interfacial Adhesion and Bulk Cohesion

Gang Lu[1,2]*, Rui Ma[3], Yuanyuan Zhao[4], Dianyu Wang[5,]*, Wentao Shang[2,6], Huaguo Chen[7], Shahid Ali Khan[2], Ming Li[8,]*, Eduardo Saiz[8]

[1] Department of Chemical and Biomolecular Engineering, University of Pennsylvania, Philadelphia, PA 19104, USA

[2] School of Energy and Environment, City University of Hong Kong, Hong Kong 999077, China

[3] NTNU Nanomechanical Lab, Department of Structural Engineering, Norwegian University of Science and Technology, Norway

[4] School of Fashion and Textile, The Hong Kong Polytechnic University, Hong Kong 999077, China

[5] School of Chemical Engineering, Zhengzhou University, Zhengzhou 450001, China

[6] Energy and Electricity Research Center, International Energy College, Jinan University, Guangdong 519070, China

[7] Department of Architecture and Civil Engineering, City University of Hong Kong, Hong Kong 999077, China

[8] Centre of Advanced Structural Ceramics, Department of Materials, Imperial College London, London, SW7 2AZ, UK

Corresponding Authors: G. Lu, E-mail: ganglu7@seas.upenn.edu
D. Wang, E-mail: wangdy@zzu.edu.cn
M. Li, E-mail: mingli24@hku.hk

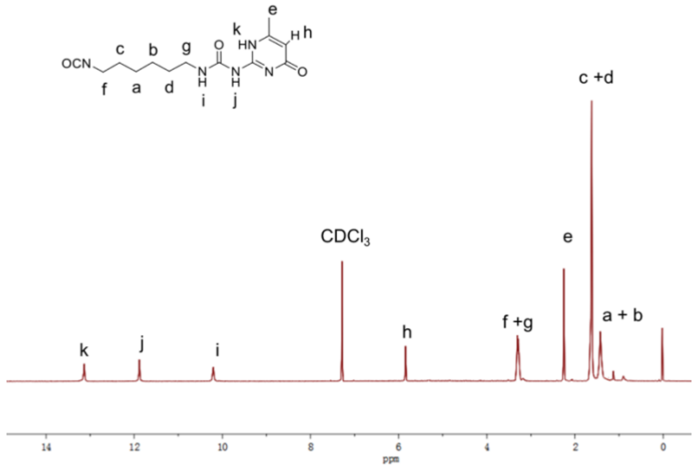

Figure S1. ^{1}H NMR spectrum of UPy-NCO recorded in $CHCl_3$-d.

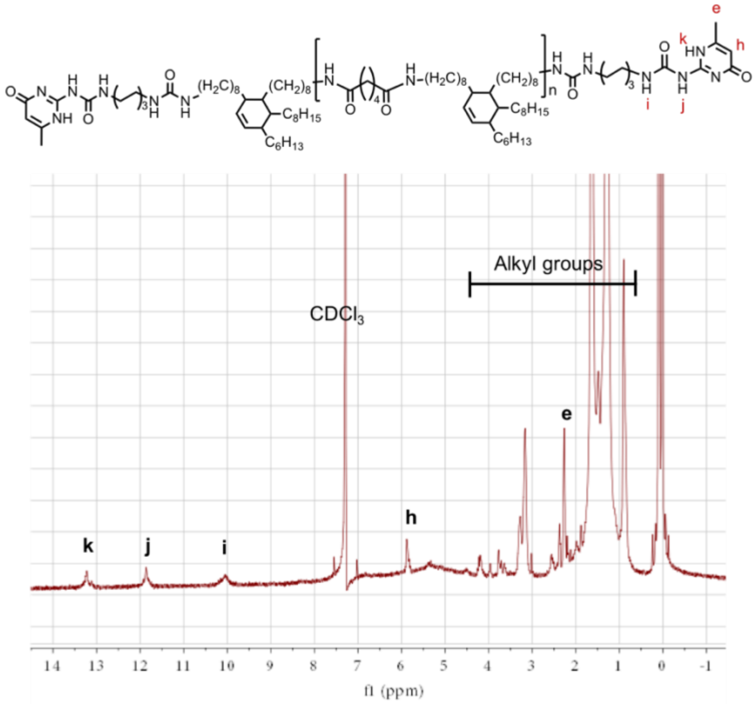

Figure S2. ^{1}H NMR spectrum of SST-2-0 recorded in $CHCl_3$-d.

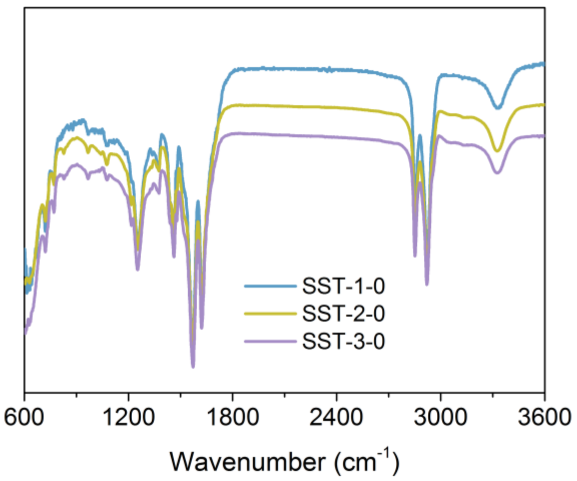

Figure S3. ATR-FTIR of SST polymers.

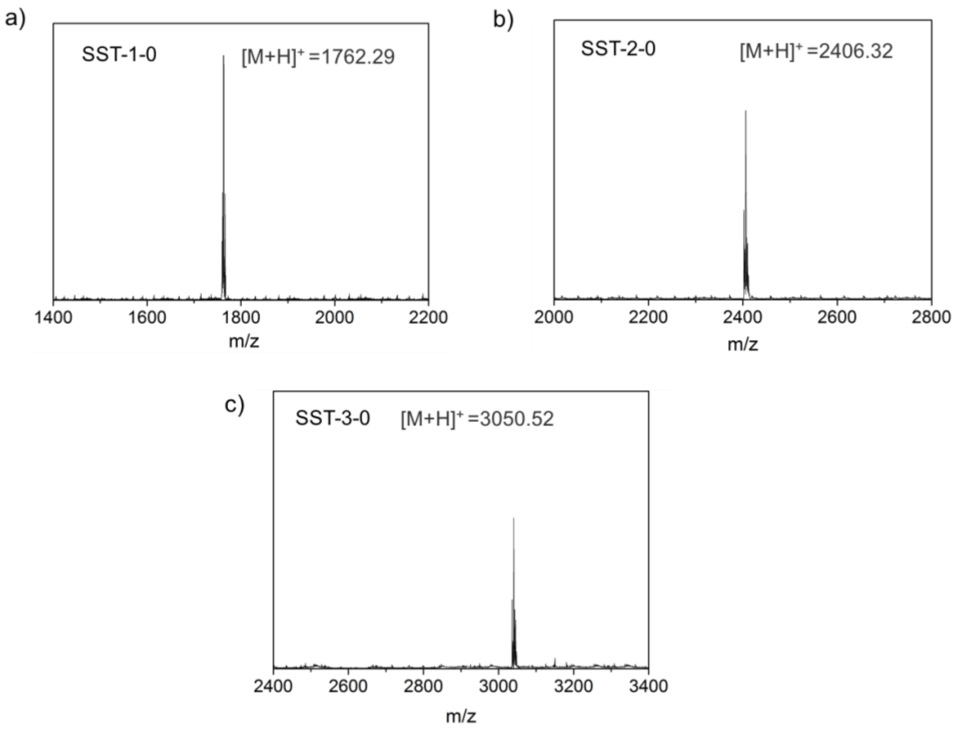

Figure S4. MALDI-TOF mass spectra using DCTB as a matrix.

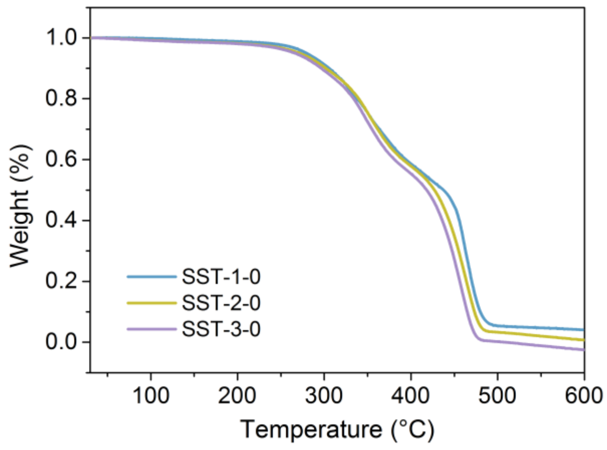

Figure S5. TGA curves of SST polymers with good thermal stability under 260°C.

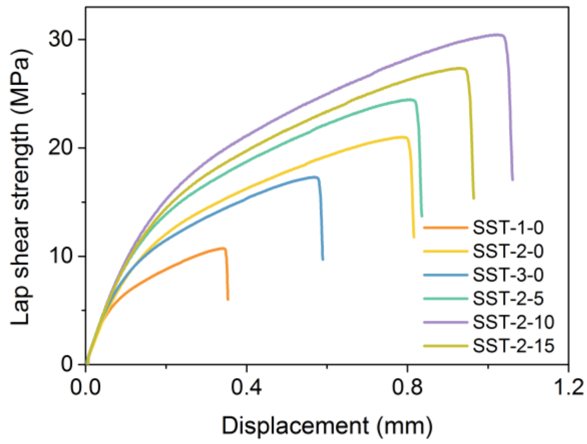

Figure S6. The lap shear curves of the sheared films.

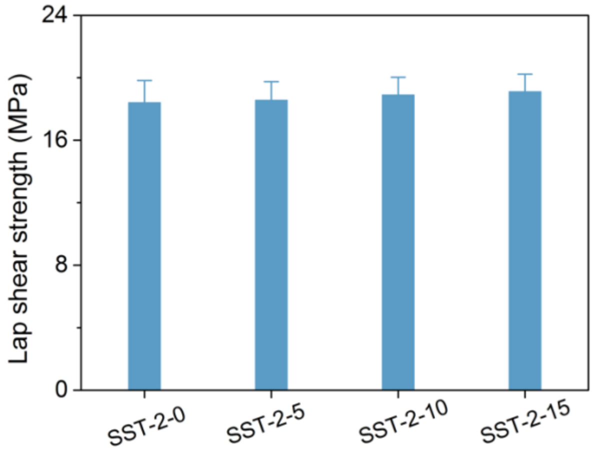

Figure S7. Lap shear stress of SST-2-n tested in a direction perpendicular to the nanofibrils. All error bars represent the SD with at least three replicates.

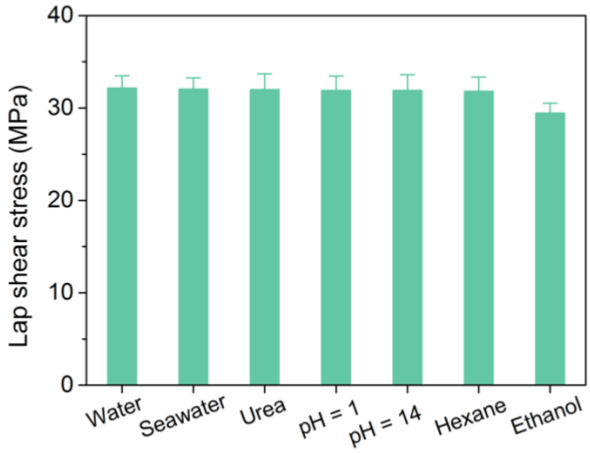

Figure S8. The lap shear strength of SST-2-10 bonded on glass substrates when treated with diverse aqueous or solvents for 24 hours. All error bars represent the SD with at least three replicates.

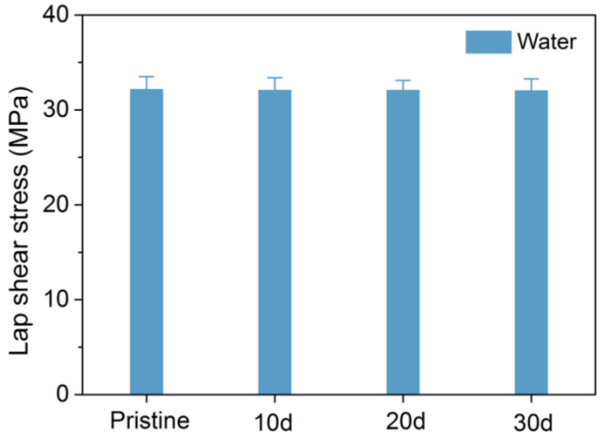

Figure S9. The lap shear strength of SST-2-10 bonded on glass substrates that were soaked in water for different time. All error bars represent the SD with at least three replicates.

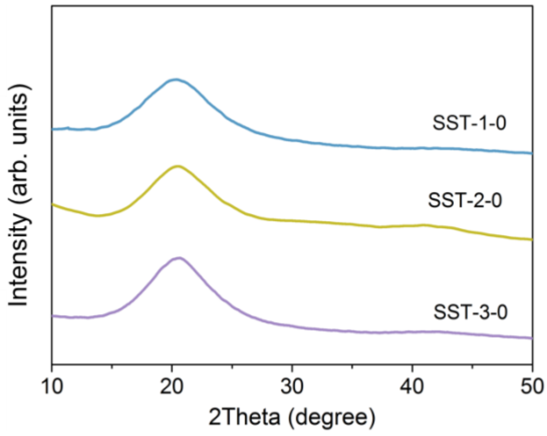

Figure S10. XRD profiles of SST-n-m prepared without solution-shearing.

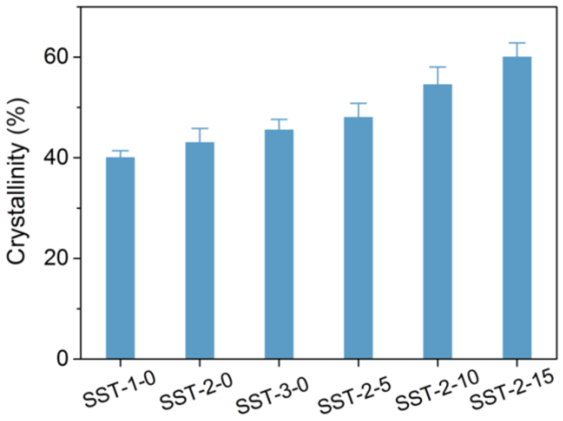

Figure S11. The crystallinity of SST polymers. All error bars represent the SD with at least three replicates.

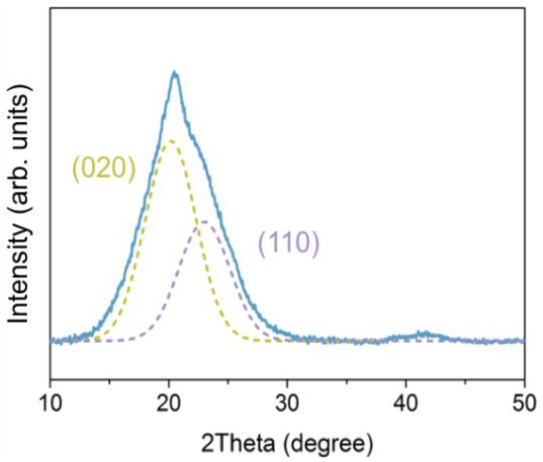

Figure S12. The deconvoluted XRD peaks of SST-2-15. The deconvoluted Peak 1 and Peak 2 in the WAXD profiles, centered at the 2 Theta of 20.18° and 23.76°, are assigned to the (020) and (110) planes of the crystalline methylene groups of adipic acid segments.

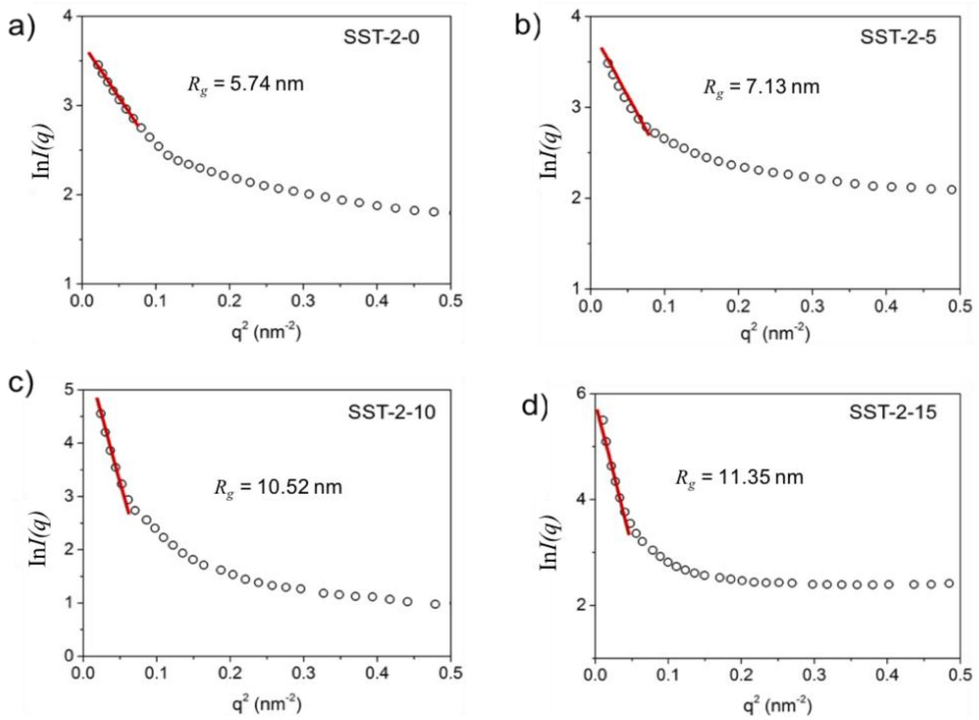

Figure S13. The gyration radius of nano-crystals of the sheared oligomers.

The radius of gyration (R_g) can be obtained from the Guinier profile [1,2]:

$$I(q) = a_0 exp\,(-\frac{R_g^2}{3}q^2)$$

where q is scattering vector, $I(q)$ is the scattering intensity, and a_0 is a constant.

Take the logarithm of Equation:

$$\ln[I(q)] = \ln a_0 - \frac{R_g^2}{3}q^2$$

Thus, R_g can be obtained from the slope of ln[$I(q)$] — q^2 curve in a small q region.

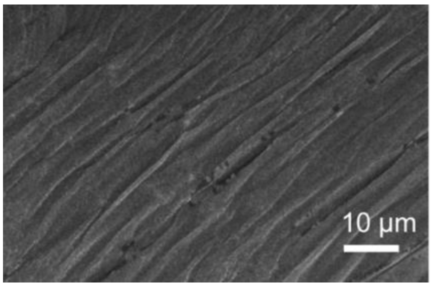

Figure S14. The formation of microfibers of SST-2-10 observed in SEM image.

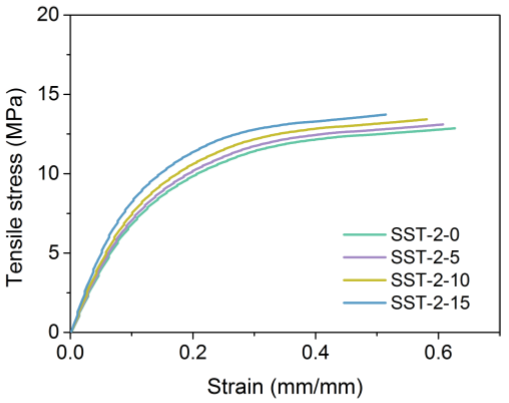

Figure S15. Tensile curves of SST-2-n tested in a direction perpendicular to the nanofibrils.

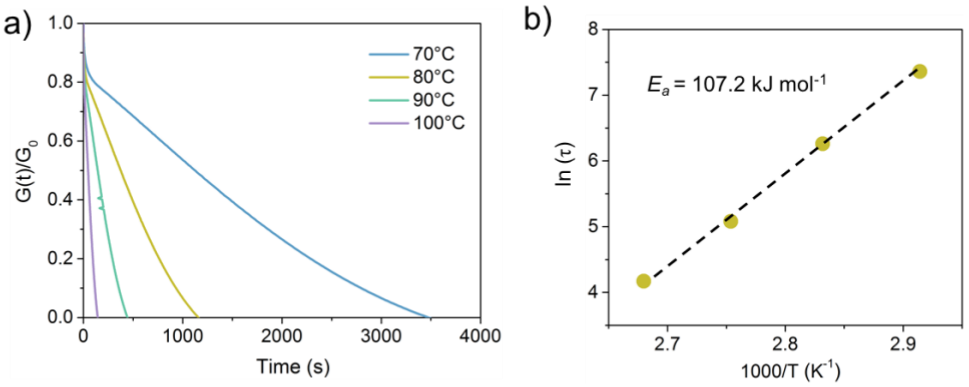

Figure S16. The temperature dependence of the relaxation time and the apparent activation energy of SST-2-0.

The temperature dependence of the relaxation time can be fitted by the Arrhenius equation [3,4]:

$$\tau(T) = \tau_0 \exp\left(\frac{E_a}{RT}\right)$$

Where τ (*T*) is relaxation time, E_a is the activation energy, and *T* is temperature.

Take the logarithm of equation:

$$\ln[\tau(T)] = \ln\tau - \frac{E_a}{RT}$$

Thus, E_a can be obtained from the slope of ln[*τ* (*T*)]-1/*T* curve.

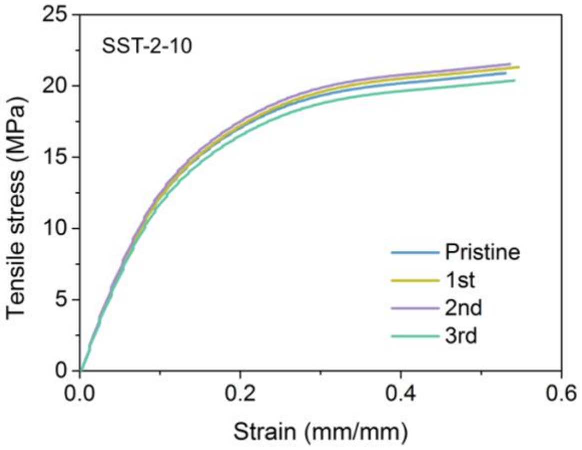

Figure S17. The tensile tests of the processed samples of SST-2-10.

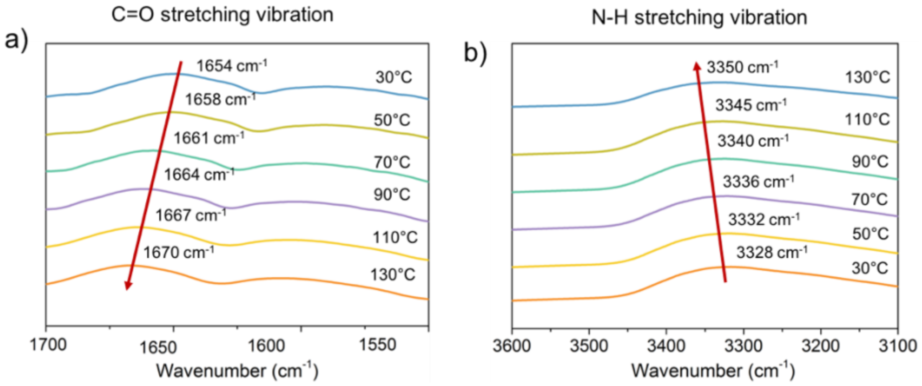

Figure S18. The stretching vibration change of C=O and NH as a function of temperature.

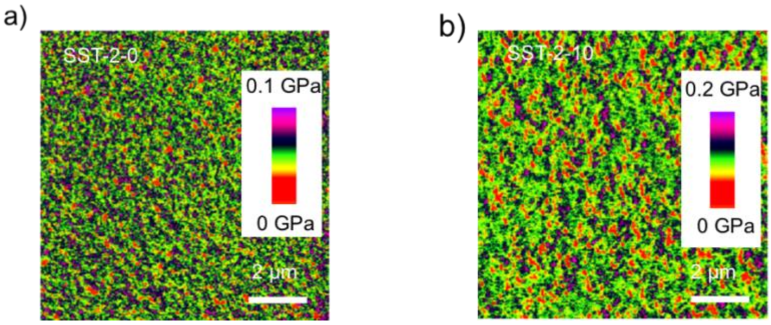

Figure S19. AFM-based surface modulus measurements of SST-2-0 and SST-2-10.

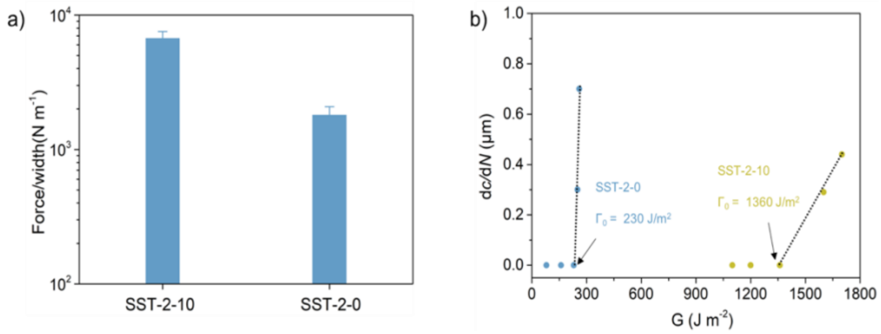

Figure S20. The measured interfacial fracture toughness (a) and interfacial fatigue threshold (b) for SST-2-10 and SST-2-0 on stainless steel substrates. All error bars represent the SD with at least three replicates.

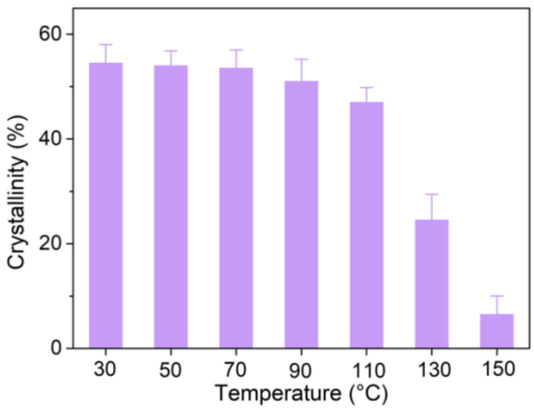

Figure S21. The crystallinity changes at elevated temperature. All error bars represent the SD with at least three replicates.

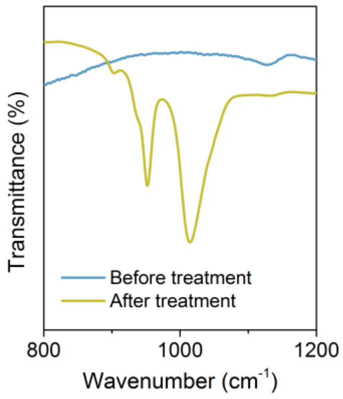

Figure S22. The sheared sample before and after heating at 60°C for 12 hours.

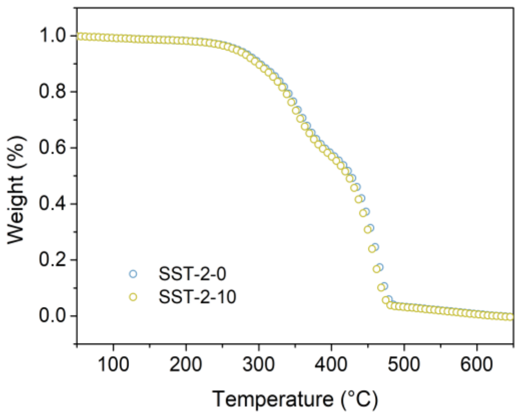

Figure S23. TGA curves of SST-2-0 and SST-2-10.

Table S1. Feed compositions of oligomers.

Compound (mmol)	SST-1-0	SST-2-0	SST-3-0
UPy-NCO	2	2	2
Priamine 1074	2	3	4
Adipic acid	1	2	3

Table S2. The mechanical properties of SST-n-m oligomers.

	Tensile strength (MPa)	Strain (mm/mm)	Young's modulus (MPa)	Toughness (MJ/m^3)
SST-1-0	15.26 ± 1.15	0.26 ± 0.01	667.32 ± 24.13	2.35 ± 0.21
SST-2-0	14.7 ± 1.07	0.72 ± 0.02	221.3 ± 24.7	4.33 ± 0.28
SST-3-0	11.43 ± 0.95	0.91 ± 0.09	116.86 ± 18.38	7.42 ± 0.26
SST-2-5	17.03 ± 1.25	0.61 ± 0.01	349.28 ± 29.69	6.73 ± 0.28
SST-2-10	20.9 ± 1.07	0.53 ± 0.02	630.52 ± 25.45	9.08 ± 0.45
SST-2-15	23.58 ± 0.98	0.22 ± 0.09	918.75 ± 28.28	6.55 ± 0.35

Table S3. Thermal properties of the sheared SST-n-m oligomers.

Compound	T_g (°C)	T_m (°C)	$\triangle H_m$ (J/g)
SST-2-0	37	138	17.2
SST-2-5	39	148	20.2
SST-2-10	41	155	23.4
SST-2-15	42	157	24.5

Table S4. Benchmarking with commercial hot melt adhesives.

Materials	Shear stress	Substrate type
3M 2665 (PU)	1.6 MPa	Stainless steel
Lubrizol 5713 (PU)	2.7 MPa	Stainless steel
Lubrizol 5714 (PU)	6.5 MPa	Stainless steel
BASF 60A (PU)	5.1 MPa	Stainless steel
Bayer 5733 (PU)	6.3 MPa	Stainless steel
Loctite 330	18.3 MPa	Stainless steel
SST-2-10	30.5 MPa	Stainless steel

Table S5. Benchmarking with commercial and reported structural adhesives[5-7].

	Materials	Shear stress	Substrate type	Source
Commercial	Poly(vinyl acetate) (Elmer's Glue All)	3.8 MPa	Aluminum	
		7 MPa	Stainless steel	
		6 MPa	Glass	
		0.48 MPa	PTFE	
	Ethyl Cyanoacrylate (Loctite Super Glue)	5 MPa	Aluminum	
		7 MPa	Stainless steel	
		6.5 MPa	Glass	
		0.7 MPa	PTFE	
	Epoxy (Loctite Quick Set)	18.5 MPa	Aluminum	
		18 MPa	Stainless steel	
		16.5 MPa	Glass	
		1 MPa	PTFE	
Reported	Benzoxazine thermoset resins	16.1 MPa	Aluminum	Angew. Chem. Int. Ed. 2019, 58, 12271
	DES-based thermosets	5.3 MPa	Glass	ACS Sustain. Chem. Eng.. 2022, 10, 41, 13816
	Polyimide thermosets	14.1 MPa	Glass	J. Mater. Chem.A, 2022, 10, 11363-11374
This work	SST-2-10	30.8 MPa	Aluminum	
		30.6 MPa	Steel	
		32.1 MPa	Glass	
		6.5 MPa	PTFE	

Table S6. Benchmarking with the reported adhesives including gels[8-10], elastomers[11-17], and resins[18-23], in terms of shear strength and work of debonding.

Type	Materials	Shear stress (MPa)	Work of debonding (kN/m)	Substrate type	Source
Gel	Polyzwitterion/clay	0.034	0.22	Glass	Adv. Mater., 2020, 32, 2004290
	Ionogel	0.43	5.48	Aluminum	Macromol. Rapid Commun., 2020, 41, 2000098
	PDMS gel	0.22	0.12	Glass	Sci. Adv., 2022, 8, eabm9744
Resin	Polypeptaides/surfactants	6.3	9.3	Steel	Angew. Chem., 2021, 60, 23687-23694
	Azobenzene derivatives	1.34	1.36	Glass	J. Am. Chem. Soc., 2019, 141, 7385-7390
	Supramolecular ionic crystal	14.6	6.32	Steel	Adv. Sci., 2022, 9, 2203182
	Polymer/dendritic crystal	3.47	1.98	Glass	Adv. Mater., 2021, 33, 2103174
	Epoxy resin	11.29	5.14	Aluminum	Biomacromolecules, 2022, 23, 779-788
	Supramolecular epoxy	10.2	6.41	Steel	ACS Mater. Lett., 2021, 3, 1003-1009
Elastomer	aWPU-30%	0.13	0.39	Glass	Adv. Funct. Mater., 2021, 31, 2007495
	PTBN6	4.21	1.11	Steel	Adv. Funct. Mater., 2022, 32, 2201959
	PTBN6	3.82	0.75	Glass	Adv. Funct. Mater., 2022, 32, 2201959
	PUIP-NAGA	7.57	7.87	Steel	Adv. Funct. Mater., 2021, 31, 2006944
	GAP-$H_{10}I_{0}$-IPDH	2.96	2.7	Steel	Chem. Eng. J., 2023, 451, 138810
	DPU-HMA	5.69	3.54	Steel	Mater. Chem. Front., 2019, 3, 1833- 1839
	Dynamic NPs	10.42	5.43	Aluminum	Sci. Adv., 2021, 7, eabk2451
	PUD20	11.37	10.32	Aluminum	Mater. Horiz., 2023, 10, 4183-4191
Solution-sheared Supramolecular oligomers	SST-2-5	24.5	15.1	Stainless steel	This work
	SST-2-10	30.6	23.6	Stainless steel	
	SST-2-15	27.4	19.4	Stainless steel	
	SST-2-10	30.8	24.1	Aluminum	
	SST-2-10	30.8	24.6	Copper	
	SST-2-10	32.2	25.3	Glass	

Table S7. Benchmarking with the reported adhesives[12,13,24-28] at elevated temperature.

Materials	Temperature	Shear stress	Substrate type	Source
SST-2-10	50°C	32 MPa	Stainless steel	This work
	70°C	33.8 MPa	Stainless steel	
	90°C	29.8 MPa	Stainless steel	
	95°C	28.7 MPa	Stainless steel	
	110°C	24.7 MPa	Stainless steel	
	120°C	21.1 MPa	Stainless steel	
	130°C	16.1 MPa	Stainless steel	
	140°C	10.1 MPa	Stainless steel	
	150°C	4.9 MPa	Stainless steel	
	110°C	24.8 MPa	Aluminum	
	90°C	30.4 MPa	Glass	
	110°C	25.5 MPa	Glass	
Epoxy (Loctite Quick Set)	95°C	2 MPa	Stainless steel	Commercial
Poly(UPy-HMDI-HEMA-co-hexyl-MA)	60°C	2.5 MPa	Stainless steel	ACS Appl. Mater. Interfaces 2015, 7, 13395–13404
Poly(UPy-HMDI-HEMA-co-butyl-MA)	60°C	4.1 MPa	Stainless steel	
20 wt % SiNPs (wet)	95°C	4.1 MPa	Stainless steel	Sci. Adv. 2021, 7, eabk2451
20 wt % SiNPs (dry)	95°C	11.4 MPa	Stainless steel	
IC gel	60°C	4.5 MPa	Glass	Angew. Chem. 2021,60,8948–8959
	90°C	0.4 MPa	Glass	
	120°C	0.1 MPa	Glass	
P(*n*BuA-*co*-Ba-*co*-HW)	60°C	12 MPa	Glass	Angew. Chem. 2022, e202203876
	90°C	6.5 MPa	Glass	
PUD20	45°C	4.2 MPa	Aluminum	Mater. Horiz., 2023, 10, 4183-4191
	60°C	1 MPa	Aluminum	
^{DC}MIN	70°C	7.1 MPa	Brass	Angew. Chem. 2024, 63, e202409705
Ionogels	85°C	0.5 MPa	Glass	J. Am. Chem. Soc. 2024, 146, 13903–13913

References

1 Liu, K. *et al.* Biomimetic impact protective supramolecular polymeric materials enabled by quadruple H-bonding. *J. Am. Chem. Soc.* **143**, 1162-1170 (2020).

2 Pan, H. *et al.* Nanoconfined crystallites toughen artificial silk. *J. Mater. Chem. B* **2**, 1408-1414 (2014).

3 Denissen, W. *et al.* Vinylogous urethane vitrimers. *Adv. Funct. Mater.* **25**, 2451-2457 (2015).

4 Yuan, W.-Q., Liu, G.-L., Huang, C., Li, Y.-D. & Zeng, J.-B. Highly stretchable, recyclable, and fast room temperature self-healable biobased elastomers using polycondensation. *Macromolecules* **53**, 9847-9858 (2020).

5 Higginson, C. J. *et al.* Bioinspired design provides high - strength benzoxazine structural adhesives. *Angew. Chem.* **131**, 12399-12407 (2019).

6 Zhao, Q. *et al.* A mussel-inspired high bio-content thermosetting polyimine polymer with excellent adhesion, flame retardancy, room-temperature self-healing and diverse recyclability. *J. Mater. Chem. A* **10**, 11363-11374 (2022).

7 Jung, S. H. *et al.* Synthesis of stimuli-responsive, deep eutectic solvent-based polymer thermosets for debondable adhesives. *ACS Sustain. Chem. Eng.* **10**, 13816-13824 (2022).

8 Gao, G. *et al.* Bioinspired self - healing human-machine interactive touch pad with pressure - sensitive adhesiveness on targeted substrates. *Adv. Mater.* **32**, 2004290 (2020).

9 Liu, Y. *et al.* Gelation of highly entangled hydrophobic macromolecular fluid for ultrastrong underwater in situ fast tissue adhesion. *Sci. Adv.* **8**, eabm9744 (2022).

10 Zhu, J., Lu, X., Zhang, W. & Liu, X. Substrate - independent, reversible, and easy - release ionogel adhesives with high bonding strength. *Macromol. Rapid Commun.* **41**, 2000098 (2020).

11 Wang, S. *et al.* Wearable stretchable dry and self - adhesive strain sensors with conformal contact to skin for high - quality motion monitoring. *Adv. Funct. Mater.* **31**, 2007495 (2021).

12 Rahman, M. A. *et al.* Design of tough adhesive from commodity thermoplastics through dynamic crosslinking. *Sci. Adv.* **7**, eabk2451 (2021).

13 Li, C. *et al.* A strain-reinforcing elastomer adhesive with superior adhesive strength and toughness. *Mater. Horiz.* **10**, 4183-4191 (2023).

14 Yang, J. *et al.* A microscale regulation strategy for strong, tough, and efficiently self-healing energetic adhesives. *Chem. Eng. J.* **451**, 138810 (2023).

15 Wang, S. *et al.* Strong, detachable, and self-healing dynamic crosslinked hot melt polyurethane adhesive. *Mater. Chem. Front.* **3**, 1833-1839 (2019).

16 Zhao, Z. H., Zhao, P. C., Zhao, Y., Zuo, J. L. & Li, C. H. An underwater long - term strong adhesive based on boronic esters with enhanced hydrolytic stability. *Adv. Funct. Mater.* **32**, 2201959 (2022).

17 Yao, Y. *et al.* Multiple H - bonding chain extender - based ultrastiff thermoplastic polyurethanes with autonomous self - healability, solvent - free adhesiveness, and AIE fluorescence. *Adv. Funct. Mater.* **31**, 2006944 (2021).

18 Wu, Z. *et al.* Green-light-triggered phase transition of azobenzene derivatives toward reversible adhesives. *J. Am. Chem. Soc.* **141**, 7385-7390 (2019).
19 Xi, S. *et al.* Reversible Dendritic‐Crystal‐Reinforced Polymer Gel for Bioinspired Adaptable Adhesive. *Adv. Mater.* **33**, 2103174 (2021).
20 Wang, W. *et al.* Double-interpenetrating-network lignin-based epoxy resin adhesives for resistance to extreme environment. *Biomacromolecules* **23**, 779-788 (2022).
21 Sun, J. *et al.* Genetically engineered polypeptide adhesive coacervates for surgical applications. *Angew. Chem.* **60**, 23687-23694 (2021).
22 Sun, P., Mei, S., Xu, J. F. & Zhang, X. A bio‐based supramolecular adhesive: ultra‐high adhesion strengths at both ambient and cryogenic temperatures and excellent multi‐reusability. *Adv. Sci.* **9**, 2203182 (2022).
23 Sun, P., Li, Y., Qin, B., Xu, J.-F. & Zhang, X. Super strong and multi-reusable supramolecular epoxy hot melt adhesives. *ACS Mater. Lett.* **3**, 1003-1009 (2021).
24 Heinzmann, C., Salz, U., Moszner, N., Fiore, G. L. & Weder, C. Supramolecular cross-links in poly (alkyl methacrylate) copolymers and their impact on the mechanical and reversible adhesive properties. *ACS Appl. Mater. Interfaces.* **7**, 13395-13404 (2015).
25 Liu, L. *et al.* A superstrong and reversible ionic crystal‐based adhesive inspired by ice adhesion. *Angew. Chem.* **133**, 9030-9041 (2021).
26 Chen, S. *et al.* Hydrogen‐Bonded Supramolecular Polymer Adhesives: Straightforward Synthesis and Strong Substrate Interaction. *Angew. Chem. Int. Ed.* **61**, e202203876 (2022).
27 Wang, Y. *et al.* Mechanically Interlocked [an] Daisy Chain Adhesives with Simultaneously Enhanced Interfacial Adhesion and Cohesion. *Angew. Chem.* **63**, e202409705 (2024).
28 Xiong, J. *et al.* Biocompatible Tough Ionogels with Reversible Supramolecular Adhesion. *J. Am. Chem. Soc.* **146**, 13903-13913 (2024).
